# A dengue-like outbreak of unknown aetiology in Pakistan

**DOI:** 10.1186/s41182-022-00426-3

**Published:** 2022-05-23

**Authors:** Hamid A. Merchant

**Affiliations:** 1 Department of Pharmacy, School of Applied Sciences, University of Huddersfield, Queensgate, Huddersfield, HD1 3DH West Yorkshire UK; 2 Health Services Academy, Chak Shahzad, Islamabad, 44000 Pakistan

**Keywords:** Dengue fever, COVID-19, Pakistan, Thrombocytopenia, Public health

## Abstract

There were recent reports of a mysterious virus spreading in Pakistan causing dengue-like illness with hundreds of cases admitted to hospitals over the winter. The mysterious virus was touted as a new variant of the dengue virus by the local health experts in Pakistan as most patients were tested negative for the dengue virus test despite presenting with dengue-like symptoms. In this letter, we have critically appraised the current situation in Pakistan in an attempt to explain the science and mechanism behind recent dengue-like outbreak, offered suggestions on diagnostic tests and provided recommendations to improve medication safety in Pakistan.

Dear Editor,

Khatri et al. recently wrote on simultaneous crises for dengue and Covid in Pakistan debilitating the country’s healthcare system [1]. In addition, there were several reports in national press of a mysterious virus causing dengue-like symptoms in Karachi with hundreds of cases admitted to hospitals over this winter [2, 7]. The mysterious virus was touted by the press as a new variant of the dengue virus as the patients tested negative for NS1 dengue antigen test, the most used diagnostic test in Pakistan for Dengue virus disease (DENV), despite patients presenting with dengue-like symptoms including thrombocytopenia and leukopenia.

It is not uncommon for viruses to mutate and evolve genetically, significant molecular changes on viral proteins can affect specificity of the molecular diagnostics. For instance, NS1 antigen tests that are commonly used in Pakistan to diagnose DENV detects the non-structural protein NS1 of the dengue virus, usually found in the patient’s blood during dengue infection. This test contains synthetically labelled antibodies that binds specifically to the NS1 proteins in blood to confirm the presence of dengue virus. Significant mutations in dengue virus would mean that the diagnostic antibodies will not be able to bind to the circulatory proteins of the dengue virus rendering NS1 test false negative. In practice, this would usually lead to the revision of the molecular diagnostics with time as the virus evolves.

It is also important to understand that NS1 is only detectable during the acute phase of the dengue virus infections. Therefore, NS1 is likely to return false negative after 7 days of symptoms. Nucleic acid amplification test (NAAT), for example PCR, should be used in this instance that is a preferred diagnostic test for dengue over NS1 antigen test, as it can provide confirmed evidence of infection in the initial phase [3]. DENV-specific IgM antibody test can also help in the diagnosis, both in first and second convalescent phase of the disease.

It is also possible that this mysterious dengue-like illness may not be dengue fever at all. Instead, this could be Covid-induced thrombocytopenia, which can often manifest clinically similar to the dengue fever. This is particularly possible in an event of coinfection with a seasonal winter bug [8] prevalent at this time of the year. Covid vaccine-induced thrombocytopenia is also a possibility that is not very well recognised and still remains significantly underreported in Pakistan [4], albeit it is a common side effect of most Covid vaccines with a frequency between 1 in 100 to 1 in 10 (1 to 10%), now also listed in vaccine's summary of product characteristics by the European Medicines Agency [5]. In most cases, vaccine-induced thrombocytopenia  resolves on its own with supportive/symptomatic care without any complications; however, in very rare instances this can manifest into more serious conditions such as immune thrombocytopenia (ITP) or vaccine-induced thrombotic thrombocytopenia (VITT) [6]. Therefore, it is important for all frontline healthcare professionals in Pakistan to be vigilant in dealing with these cases of dengue-like illness and offer appropriate interventions to these patients as the case may be.

The healthcare professionals in Pakistan are also urged to report all suspected adverse events following Covid vaccines to the National Pharmacovigilance Centre in Pakistan to help with vaccines pharmacovigilance to support global regulatory and public health agencies in  improving medication safety.

## Data Availability

Not applicable.
